# Clinical Association of Haptoglobin with Oxidized LDL in Obese Patients with Type 2 Diabetes Mellitus

**DOI:** 10.3390/nu17172883

**Published:** 2025-09-06

**Authors:** Ahmed Bakillah, Maram Al Subaiee, Khamis Khamees Obeid, Ayman Farouk Soliman, Abeer Al Otaibi, Sindiyan Al Shaikh Mubarak, Yara Abdullah Al Mihmadi, Shahinaz Faisal Bashir, Mohammad Al Arab, Arwa Al Hussaini, Ali Ahmed Al Qarni

**Affiliations:** 1 King Abdullah International Medical Research Center (KAIMRC), Al Ahsa 36428, Saudi Arabia; otaibiabe@kaimrc.edu.sa (A.A.O.); alshaikhmubaraks@kaimrc.edu.sa (S.A.S.M.); mihmadiy@kaimrc.edu.sa (Y.A.A.M.); bashirsha@kaimrc.edu.sa (S.F.B.); alhussainiar@kaimrc.edu.sa (A.A.H.); qarniaa@mngha.med.sa (A.A.A.Q.); 2 Biomedical Research Core, King Saud bin Abdulaziz University for Health Sciences (KSAU-HS), Al Ahsa 36428, Saudi Arabia; 3 King Abdulaziz Hospital, Ministry of National Guard Health Affairs (MNGHA), Al Ahsa 36428, Saudi Arabia; alsubaieema@mngha.med.sa (M.A.S.); khamisko@mngha.med.sa (K.K.O.); solimana@mngha.med.sa (A.F.S.)

**Keywords:** haptoglobin, oxidized LDL, type 2 diabetes mellitus, obesity, cardiovascular risk, kidney diseases, atherosclerosis, urinary biomarkers, insulin resistance, inflammation, oxidative stress, endothelial dysfunction

## Abstract

**Background**: Cardiovascular disease (CVD) is the leading cause of mortality in obese patients with type 2 diabetes mellitus (T2DM). Conventional biomarkers often fail to detect early endothelial dysfunction and oxidative stress. Haptoglobin (Hp), an acute-phase protein with antioxidant and hemoglobin-binding properties, may indicate vascular injury. While plasma Hp (pl-Hp) reflects systemic inflammation, urinary Hp (u-Hp) could signal renal and microvascular damage. We hypothesize that elevated u-Hp and altered pl-Hp levels are associated with increased oxidized LDL and may serve as sensitive indicators of early vascular injury, thereby identifying obese patients with T2DM at higher cardiovascular risk. This study aims to investigate the associations between u-Hp, pl-Hp, and oxidized LDL (ox-LDL) in obese patients with T2DM, and to evaluate the potential role of Hp as an early biomarker of cardiovascular risk in this high-risk population. **Methods and Results**: The study included 57 patients with T2DM (mean age 61 ± 10 years, HbA1c 8.66 ± 1.60%, and BMI 35.15 ± 6.65 kg/m^2^). Notably, 95% of the patients had hypertension, 82% had dyslipidemia, and 59% had an estimated glomerular filtration rate (eGFR) < 60 mL/min/1.73 m^2^. Pl-Hp and u-Hp concentrations, as well as ox-LDL levels, were assessed using an enzyme-linked immunosorbent assay (ELISA). Correlations and multivariate regression analyses were employed to investigate the associations between Hp, ox-LDL, and clinical cardiovascular risk factors. Pl-Hp was positively correlated with ox-LDL (r = 0.358, *p* < 0.006) and negatively correlated with C-reactive protein (CRP) (r = −0.364, *p* < 0.013), while u-Hp correlated positively with HbA1C and apoB levels (r = 0.298, *p* < 0.030 and r = 0.310, *p* < 0.021, respectively). Multivariate analysis indicated that pl-Hp, but not u-Hp, was independently associated with ox-LDL (β = 0.536, *p* < 0.027) after adjusting for potential confounding factors, including age, gender, BMI, HbA1c, liver enzymes, hs-CRP and creatinine. The Stepwise analysis identified IL-6 as the most significant predictor of cardiovascular disease risk, suggesting its pivotal role in subclinical vascular inflammation among obese individuals with T2DM. Furthermore, the significant positive association between pl-Hp and ox-LDL was stronger in patients with declining renal function as expressed by the estimated glomerular filtration rate (eGFR) (eGFR < 30 mL/min/1.73 m^2^: β = 2.173, *p* < 0.031 and eGFR 30–59 mL/min/1.73 m^2^: β = 1.318, *p* < 0.002). This association also appeared in early and low-normal ranges of serum albumin: creatinine ratio (s-ACR) (s-ACR < 0.2714 mg/mmol: β = 2.304, *p* < 0.005 and s-ACR 0.2714–0.3649 mg/mmol: β = 1.000, *p* < 0.041), suggesting that pl-Hp and ox-LDL rise before overt kidney damage. Elevated IL-6 (≥32.93 pg/mL) further strengthened this link (β = 1.037, *p* < 0.005), highlighting the role of inflammation in amplifying oxidative stress and acute-phase responses. **Conclusions**: Taken together, these findings emphasize the interconnected contributions of renal impairment, inflammation, and oxidative stress to vascular injury. While these results need to be confirmed in larger prospective longitudinal studies, monitoring pl-Hp levels in conjunction with inflammatory and kidney function markers could be a sensitive and non-invasive way to identify early CVD risk in high-risk groups, such as obese patients with T2DM.

## 1. Introduction

Obesity and type 2 diabetes mellitus (T2DM) are significant global health challenges that frequently coexist and synergistically elevate the risk of cardiovascular disease (CVD) [1,2]. These metabolic disorders are marked by chronic low-grade inflammation, oxidative stress, and endothelial dysfunction, which are the hallmarks of the atherosclerotic process [3]. Timely identification of non-invasive biomarkers reflecting these underlying pathophysiological processes is essential for the early detection and prevention of cardiovascular complications in high-risk populations.

Haptoglobin (Hp) is an acute-phase glycoprotein (~85 kDa), predominantly synthesized by the liver, that binds free hemoglobin (Hb) to prevent heme-mediated oxidative damage and preserve iron homeostasis [4,5]. While Hp does not bind oxidized low-density lipoprotein (ox-LDL) directly, it indirectly modulates oxidative stress by limiting Hb-induced lipid peroxidation [6,7,8]. This protective function may influence macrophage activation, foam cell formation, and ultimately atherogenesis [9].

Genetic polymorphisms in the Hp gene result in three major phenotypes, Hp1-1, Hp2-1, and Hp2-2, which differ in their antioxidant potential [10]. The Hp2 allele is generally more frequent worldwide, especially in African and Middle Eastern populations, while East Asians tend to have a higher frequency of the Hp1 allele [11]. Complete haptoglobin deficiency, Anhaptoglobinemia, is rare, mainly found in East Asian populations at low frequencies, and is usually asymptomatic [11]. Hp polymorphisms influence the protein’s ability to bind free hemoglobin and modulate oxidative stress. The Hp1-1 genotype offers the strongest antioxidant protection [12]. In contrast, the Hp2-2 genotype is less effective and has been associated with increased oxidative stress, higher ox-LDL accumulation, and a significantly elevated risk of CVD in individuals with diabetes [13,14,15,16,17,18,19,20,21,22,23,24]. Translational studies further illustrate that oxidative stress in Hp2-2 diabetic subjects impairs reverse cholesterol transport. At the same time, the prolonged circulation of the Hp–Hb complex and iron loading within the atherosclerotic plaques contribute directly to endothelial and vascular injury [4,25,26,27].

In states of metabolic dysfunction, such as obesity and T2DM, plasma haptoglobin (pl-Hp) levels are typically elevated as part of the acute-phase hepatic response to inflammation and increased hemolysis [28,29,30]. Elevated pl-Hp has been linked to vascular inflammation and oxidative stress, including increased circulating ox-LDL levels [19,29,30,31]. This association is particularly pronounced among individuals with the Hp2-2 phenotype, supporting its potential utility in cardiovascular risk stratification.

Urinary haptoglobin (u-Hp) has recently emerged as a potential biomarker for renal and microvascular injury [24,32,33,34]. Under physiological conditions, Hp is rarely detectable in urine because of its size and efficient reabsorption in the proximal tubules. However, in the context of diabetic nephropathy or systemic inflammation, the excretion of u-Hp increases, reflecting glomerular damage or impaired tubular function. The urinary Hp-to-creatinine ratio (u-HCR) has been proposed as a promising early marker of renal injury, particularly in obese individuals with T2DM, where it may precede traditional indicators such as albuminuria [33,34,35,36]. While pl-Hp reflects systemic inflammation and oxidative stress, u-Hp may serve as a more sensitive marker of renal endothelial dysfunction and microvascular damage, both of which are critical contributors to cardiovascular risk in T2DM [32,34]. Recent findings suggest that both pl-Hp and u-Hp are independently associated with elevated ox-LDL levels, reinforcing their complementary roles as indicators of cardiovascular risk [27,37,38,39]. However, the interplay between pl-Hp, u-Hp, and ox-LDL has not been systematically examined in obese patients with T2DM. Addressing this gap is essential to better understand whether HP can serve as a practical biomarker of cardiovascular risk in this population.

Despite growing interest in Hp as a dual-biomarker, the relative clinical utility of plasma versus urinary Hp remains unclear [24,40,41]. Each one reflects distinct pathological pathways through various regulatory mechanisms across compartments. Assessing simultaneously both markers may offer a more comprehensive picture of cardiometabolic risk by capturing both systemic and organ-specific oxidative injury.

We hypothesize that elevated u-Hp and altered pl-Hp levels are associated with increased ox-LDL and may serve as sensitive indicators of early vascular injury, thereby identifying obese patients with T2DM at higher cardiovascular risk. Therefore, in this study, we aimed to investigate the relationship between Hp and ox-LDL levels, as well as to assess whether pl-Hp functions as an independent predictor of cardiovascular risk beyond traditional clinical markers in obese patients with T2DM. If validated, pl-Hp could be incorporated into cardiovascular risk assessment models for obese patients with T2DM, leading to earlier interventions, more targeted therapies, and ultimately reduced cardiovascular events in this high-risk group.

## 2. Materials and Methods

### 2.1. Study Population and Protocol

This retrospective study involved 57 patients with T2DM recruited from clinics at King Abdulaziz Hospital (KAH), Ministry of National Guard-Health Affairs in Al-Ahsa, Kingdom of Saudi Arabia, between January 2020 and April 2021. The study protocol received approval from the Institutional Review Board of the Ministry of National Guard, Health Affairs (IRB protocol# IRBC/1972/18), and written informed consent was obtained from each participant. Participants were excluded from the study if, at baseline, they met one or more of the following criteria: being on chronic renal replacement therapy (such as hemodialysis, peritoneal dialysis, or transplantation), having a history of active malignancy (excluding basal cell carcinoma) within the last five years (or prostatic cancer within the past two years), systemic lupus erythematosus, and other autoimmune diseases affecting kidney function, a history of type-1 diabetes, acute infection or fever, pregnancy, chronic viral hepatitis or HIV infection, or current unstable cardiac disease. Standard definitions were applied as follows: Diabetes: history of T2DM on medication, HbA1c ≥ 6.5%, or fasting glucose ≥ 126 mg/dL (≥7 mmol/L). Family history of T2DM: any first-degree relative with T2DM. Dyslipidemia: history of dyslipidemia on medication, total cholesterol > 200 mg/dL, or LDL > 70 mg/dL. Hypertension: systolic BP ≥ 140 mmHg, diastolic BP ≥ 90 mmHg, or use of antihypertensive therapy. CKD: eGFR < 90 mL/min (MDRD equation) or proteinuria (≥2+ on dipstick).

### 2.2. Measurement of Haptoglobins and Ox-LDL Levels in T2DM Patients

Fasting blood samples were collected in the morning after a minimum of 12 h of fasting, placed into tubes containing EDTA, and centrifuged at 4 °C at 3000 rpm for 10 min to separate the plasma for biochemical tests. Samples from patients with T2DM were aliquoted and stored at −80 °C until further analysis. Patient medical history, demographics, and laboratory parameters were extracted from the electronic medical record in the BEST Care database. Concentrations of plasma and urinary Hp and ox-LDL were determined by ELISA using commercially available kits according to the manufacturer’s protocols (catalog# BMS2880564 from MyBioSource, San Diego, CA, USA, and E-EL-H6021 from Elabscience Biotechnology Inc., Houston, TX, USA; respectively). The ox-LDL kit has a sensitivity of 37.5 pg/mL, with a detection range of 62.5 to 4000 pg/mL, according to the manufacturer. In contrast, the Hp kit has a sensitivity of less than 1.56 ng/mL, with a detection range of 3.12–200 ng/mL. Adipokine and cytokine levels were measured using the multiplex^®^ MAP Human Adipokine Magnetic Bead Panels 1, 2, and 3 (catalogs# HADK1MAG-61K, HADK2MAG-61K, and HCVD3MAG-67; EMD Millipore-Sigma; Burlington, MA, USA). Human adiponectin/ACRP30 concentrations were assessed using a commercially available ELISA kit (catalog # E-EL-H6122 from Elabscience Biotechnology Inc., Houston, TX, USA).

### 2.3. Statistical Analysis

Statistical analyses were performed using SPSS software version 30.0 (IBM Corp., Chicago, IL, USA. The normality of the data was evaluated using the Kolmogorov–Smirnov test. Continuous variables that showed a normal distribution were presented as means ± standard deviation (SD), while categorical variables were reported as frequencies and percentages. We utilized the non-parametric Spearman’s correlation test to assess the correlation between Hp, ox-LDL, and other variables. We conducted a multiple regression analysis to investigate the association between urinary and Pl-Hpand ox-LDL, adjusting the model for variables including age, sex, HbA1c, creatinine, BMI, total cholesterol, LDL-C, HDL-C, and triglycerides. Multivariate analyses were adjusted for key medications, including statins, antihypertensives, and insulin therapy, to account for their potential confounding effects on oxidative stress, inflammation, and biomarker levels. A stepwise analysis was performed to identify the optimal subset of predictor variables influencing the association between Hp and ox-LDL. Regression analysis results were reported as β-coefficients and 95% confidence intervals. For statistical significance, two-sided tests with *p*-values < 0.05 were considered significant.

## 3. Results

### 3.1. Baseline Characteristics of the Study Subjects with T2DM

Table 1 summarizes the characteristics of the study subjects with T2DM. The patients had a mean age of 60.96 ± 9.99 years, with 44% being female. All subjects were Saudi citizens, and 82% had a family history of diabetes, while 58% had a family history of cholesterol issues. A majority of the subjects (95%) had hypertension, 79% had dyslipidemia, and 14% had coronary artery disease. The mean HbA1c level was 8.66% ± 1.60%. Most subjects were overweight, with a mean BMI of 35.15 ± 6.65 kg/m^2^. The median serum creatinine level was 111.50 µmol/L (81.00–142.25), which exceeds the typical reference range for adults (~60–110 µmol/L for males and 45–90 µmol/L for females), indicating possible reduced renal clearance. Furthermore, the eGFR was 58.04 ± 26.50 mL/min/1.73 m^2^, which falls within the range of chronic kidney disease stage 3 (moderate reduction in kidney function), suggesting that a significant proportion of participants have underlying renal impairment.

While there is currently no standardized reference range for u-Hp, the median value of u-Hp in our cohort was 10.45 ng/mL (range: 3.58–73.13 ng/mL), which is lower than the reported median u-Hp concentration of 70 ng/mL in the study of 204 participants from the Veterans Affairs Diabetes Trial (VADT) [32]. In contrast, the median value of pl-Hp was 4,105,900 ng/mL (range: 2,456,100–5,906,600 ng/mL), which is above the normal range in adults (300,000–2,000,000 ng/mL) [42,43]. Additionally, ox-LDL levels were elevated, with a mean value of 247.97 ± 67.50 ng/mL, compared to the established reference range of 10–170 ng/mL for ox-LDL [44,45]. Circulating pro-inflammatory cytokines and adipokines were elevated, reflecting a systemic inflammatory and metabolic dysregulation profile characteristic of individuals with metabolic disorders, such as T2DM and/or obesity-related conditions. IL-6 and TNF-α demonstrated median levels of 24.72 pg/mL and 38.80 pg/mL, respectively, which are notably higher than levels typically reported in healthy individuals (generally <10 pg/mL for both). This elevation suggests an ongoing low-grade chronic inflammatory state that may contribute to insulin resistance, endothelial dysfunction, and lipid abnormalities. Adiponectin was measured at 3735.74 ± 966.21 ng/mL. While this falls within a moderate range, it is relatively low given adiponectin’s anti-inflammatory and insulin-sensitizing roles. Additionally, Resistin levels were highly elevated (median: 46,889.39 pg/mL), supporting its role as a pro-inflammatory adipokine that may further exacerbate insulin resistance and cardiovascular risk. Similarly, Adipsin exhibited a wide range, with a median of 583,813.15 pg/mL, potentially reflecting compensatory immune-metabolic responses, given its role in complement system activation and regulation of β-cell function.

### 3.2. Correlations Between Urinary and Plasma Haptoglobin with Ox-LDL Levels and Clinical Parameters in T2DM Patients

Spearman correlation analysis indicated that u-Hp showed a positive correlation with HbA1c and apoB levels (r = 0.298, *p* = 0.030 and r = 0.310, *p* = 0.021, respectively; Table 2), while pl-Hp exhibited a positive correlation with ox-LDL (r = 0.358, *p* = 0.006; Table 2) and a negative correlation with CRP (r = −0.364, *p* = 0.013; Table 2). Furthermore, there were no significant correlations between u-Hp and pL-Hp, nor with other parameters, including age, gender, e-GFR, hypertension, total cholesterol, LDL-c, HDL-c, triglycerides, Hs-CRP, and BMI (Table 2).

### 3.3. Univariate Regression Analysis for the Association of Urinary and Plasma Haptoglobin with Ox-LDL in T2DM Patients

We first conducted a univariate analysis to examine the association between urinary and plasma levels of Hp and Ox-LDL. The results revealed a positive association between pl-Hp and ox-LDL (β = 0.741, *p* = 0.002, r^2^ = 0.393; Table 3). In contrast, there was no significant association between u-Hp and ox-LDL (β = 0.038, *p* = 0.585; Table 3).

### 3.4. Multivariate Regression Analysis for the Association Between Plasma Haptoglobin with Ox-LDL in T2DM Patients

We examined whether additional factors could help clarify the relationship between pl-Hp and ox-LDL levels. To do this, we performed a multiple regression analysis, adjusting for a range of potential confounders, including age, sex, HbA1c, creatinine, total cholesterol, LDL-C, HDL-C, triglycerides, hs-CRP, BMI, ALT, AST, as well as various adipokines and cytokines (Table 4). The analysis showed that pl-Hp remained independently associated with ox-LDL (Model 3: β = 0.678; *p* = 0.023), even after controlling for these variables. In contrast, pl-Hp was not independently associated with ox-LDL (Model 4: B = 0.028, *p* = 0.177; Table 4) after adjusting for adipokines/cytokines and inflammatory markers. Significant predictors of ox-LDL included hsCRP (β = −0.542, *p* = 0.018), adiponectin (β = −0.556, *p* < 0.001), and resistin (β ≈ 0.000, *p* = 0.047); Table 4.

### 3.5. Stepwise Regression Analysis for the Identification of the Best Predictors for the Association Between Plasma Haptoglobin and Ox-LDL in T2DM Patients

Additionally, we used stepwise linear regression to examine how circulating inflammatory biomarkers (hs-CRP and IL-6) and kidney function and damage biomarkers (e-GFR, u-ACR, and s-ACR) affect the relationship between pl-Hp and Ox-LDL. This analysis enabled us to identify the optimal subsets of potential predictors for the relationship between Pl-Hp and Ox-LDL (Table 5). We added independent variables from the selected biomarkers to the regression models (M1–M2) along with Pl-Hp. The results showed that IL-6 had the most significant impact when included in the predictive model, explaining 27% of the variability in the relationship between Pl-Hp and Ox-LDL (β = 0.680, *p* = 0.006, M2; Table 5).

### 3.6. Multiple Regression Analysis for the Association Between Plasma Haptoglobin and Ox-LDL Across Categorized Subgroups in T2DM Patients

To explore how the connection between pl-Hp and ox-LDL may change in response to systemic inflammation or alterations in kidney function, we aimed to better understand the relationships among oxidative stress, inflammation, and kidney issues. For this purpose, we employed multiple regression analysis to investigate the relationship between pl-Hp and ox-LDL across various groups defined by biomarkers for inflammation and kidney function. We examined interactions within subgroups categorized by levels of hs-CRP, IL-6, eGFR, u-ACR, and s-ACR (Table 6). The multiple regression analysis revealed a significant association between pl-Hp and ox-LDL among patients with a moderate decrease in kidney function (e-GFR = 30–59 mL/min/1.73 m^2^; Table 6) and those with severe decrease or kidney failure (e-GFR < 30 mL/min/1.73 m^2^; Table 6). Furthermore, the association between pl-Hp and ox-LDL remained significant among patients with IL-6 levels greater than 32.93 pg/mL and s-ARC levels below 0.36 mg/mmol (Table 6). In contrast, there was no significant association between pl-Hpand ox-LDL across all categorized groups for u-ACR (Table 6).

## 4. Discussion

Obesity and T2DM are closely associated with an increased risk of cardiovascular disease, primarily driven by chronic low-grade inflammation and oxidative stress [46]. A key component in this oxidative state is ox-LDL, which contributes to endothelial dysfunction, atherosclerosis, and continuous vascular damage [47]. Hp, an acute-phase protein with antioxidant effects, plays a crucial role in neutralizing free Hb and reducing oxidative harm. However, variations in Hp levels and function, particularly in metabolic disorders, may influence ox-LDL accumulation and its detrimental effects. Although there is increasing interest in using Hp as a biomarker for cardiovascular risk among obese individuals with T2DM, its clinical usefulness remains uncertain. Most research has either focused on genetic factors or analyzed Hp alone, without considering the broader context of inflammatory and oxidative status [19,48,49,50]. By studying the interactions between Hp and ox-LDL, we offer a comprehensive evaluation of both plasma and urinary Hp in high-risk obese individuals with T2DM, exploring how Hp can be integrated into biomarker panels for early CVD risk prediction and clinical application.

Previous reports indicated significant increases in Hp and ox-LDL levels in cardiovascular and metabolic diseases, including diabetes and obesity [35,51,52,53]. Compared to published reports on established value reference ranges in a healthy population (ox-LDL < 50 ng/mL, u-Hp < 0.01 ng/mL and Pl-Hp = 300,000–2,000,000 ng/mL), our study revealed that circulating levels of ox-LDL (247.97 ng/mL), u-Hp and pl-Hp levels (10.45 ng/mL and 4,159,000 ng/mL; respectively) were significantly elevated among our cohort’s subjects, which aligns with previously published studies [29,34,35,54,55].

In normal conditions, Hp is not freely filtered through the kidney glomerular basement membrane due to its large size and remains bound to Hb in the plasma, forming an even larger complex (>150 kDa). In contrast, under pathological states such as diabetic nephropathy and hypertension, where the glomerular filtration barrier is damaged, large proteins, including Hp, escape from circulation and pass into the filtrate. Notably, a positive correlation was observed between pl-Hp and ox-LDL, but not with u-Hp. Furthermore, there was no significant correlation between u-Hp and pl-Hp. The lack of correlation between plasma and urinary Hp supports the notion that u-Hp is a marker of localized renal injury rather than a spillover product from plasma. These findings underscore the value of evaluating plasma and urinary Hp separately, as they could provide complementary insights into systemic oxidative stress and kidney-specific damage, respectively.

Additionally, our findings demonstrate a significant positive correlation between u-Hp levels and HbA1c in obese patients with T2DM, whereas no such relationship was observed with pl-Hp. This divergence likely reflects the distinct physiological roles and regulatory mechanisms of Hp in systemic circulation versus the renal environment. Pl-Hp is primarily synthesized by the liver in response to systemic inflammation, oxidative stress, and multiple factors, including hemolysis, infection, and genetic polymorphisms, that influence its levels. As such, pl-Hp lacks specificity for hyperglycemia-related tissue injury. In contrast, u-Hp is known to serve as a sensitive marker of glomerular and proximal tubular dysfunction, particularly in the early stages of diabetic kidney disease. Chronic hyperglycemia, as indicated by elevated HbA1c, progressively impairs the glomerular filtration barrier and proximal tubular reabsorption capacity, enabling the abnormal appearance and accumulation of Hp in urine. Therefore, the observed positive correlation between u-Hp and HbA1c likely reflects the cumulative burden of hyperglycemia-induced renal injury, supporting the utility of u-Hp as an early biomarker of diabetic nephropathy.

Interestingly, we observed an unexpected inverse relationship between Hp and CRP, although both are acute-phase reactants. This paradoxical association has been observed under specific pathophysiological conditions; however, further studies are required to confirm this observation [56,57]. This paradoxical relationship may reflect the complex and divergent regulatory pathways governing the expression and metabolism of these two markers in chronic metabolic inflammation. CRP synthesis in hepatocytes is strongly induced by IL-6 and synergistically enhanced by IL-1β, whereas Hp regulation appears more dependent on IL-6 and modulated by other cytokines, including IL-10. This suggests that variations in cytokine profiles may differentially affect the two proteins, leading to paradoxical associations. In addition, Hp is primarily involved in hemoglobin binding and clearance. In contrast, CRP is a pattern-recognition protein that rapidly rises in acute inflammation but can also decline quickly once the stimulus resolves. Medication effects, particularly statins and insulin therapy, may further contribute to these divergent responses by modulating inflammatory signaling [58]. While Hp is also upregulated during inflammation, its plasma levels are strongly influenced by oxidative stress, hemolysis, and genetic polymorphisms, and it may be subject to depletion or consumption in chronic pathological conditions. In T2DM, persistent oxidative stress and low-grade hemolysis can accelerate the clearance of Hp through its binding to Hb, reducing its circulating levels despite ongoing inflammation [29,59]. Moreover, hepatic dysfunction associated with insulin resistance may alter the acute-phase protein production profile, favoring CRP synthesis over Hp. Thus, the inverse correlation observed in this study likely reflects the consumption or insufficient production of Hp in the setting of elevated inflammatory load, as captured by CRP, highlighting the nuanced and non-redundant roles of these two markers in chronic metabolic disease. This finding should be interpreted cautiously and considered hypothesis-generating. Future studies incorporating hemolysis markers, liver function assessment, and Hp genotyping are needed to elucidate the underlying biology.

Our study revealed a significant positive association between pl-Hp and ox-LDL levels, while no correlation was observed with u-Hp. This finding highlights the distinct pathophysiological domains reflected by circulating versus urinary Hp. As an acute-phase protein with antioxidant properties, pl-Hp plays a key role in neutralizing free Hb and limiting oxidative damage, including the propagation of lipid peroxidation that leads to ox-LDL formation. Elevated pl-Hp levels may therefore reflect a systemic compensatory response to increased oxidative stress, which is commonly observed in obesity and T2DM [28,29,60]. In this context, higher pl-Hp concentrations may coexist with elevated ox-LDL levels as both are upregulated in response to the same underlying oxidative milieu. Notably, pl-Hp is independently associated with ox-LDL concentrations, even after adjusting for demographic, clinical, and metabolic covariates. These results suggest that systemic pl-Hp levels may more directly reflect or participate in oxidative lipoprotein modification processes, particularly in the context of obesity and T2DM.

Obese individuals with T2DM often exhibit dysregulated adipokine profiles, including reduced adiponectin and elevated resistin levels, along with increased concentrations of pro-inflammatory cytokines such as IL-6 and TNF-α [61,62]. These mediators are known to influence both oxidative pathways and acute-phase responses, thereby potentially modulating Hp expression and function. In the multivariable models, the link between pl-Hp and ox-LDL was weakened and became nonsignificant after adjusting for inflammatory and metabolic covariates. Instead, ox-LDL levels were independently predicted by hs-CRP, adiponectin, and resistin. These results indicate that pl-Hp reflects systemic inflammatory activity but does not independently influence ox-LDL levels once key mediators of inflammation and adipokine balance are considered. This highlights the complex interplay between acute-phase proteins, oxidative stress, and adipokine signaling in the context of obesity and T2DM.

Additionally, cohort subgroups analysis revealed that the positive association between pl-Hp and ox-LDL was especially evident in individuals with compromised renal function. In participants with eGFR between 30–59 mL/min/1.73 m^2^, representing moderate CKD (stage 3), the regression analysis showed a significant association (β = 1.318, *p* < 0.002). Moreover, in patients with eGFR < 30 mL/min/1.73 m^2^ (CKD stage 4–5), this relationship was even stronger (β = 2.173, *p* < 0.031), suggesting that worsening kidney function may amplify oxidative stress and trigger a heightened acute-phase response. This could be due to reduced renal clearance of circulating Hp and ox-LDL, increased hemolysis, or persistent systemic inflammation characteristic of advanced CKD [63]. These findings support the notion that renal dysfunction is not only a consequence of but also a driver of oxidative-inflammatory interactions.

Similarly, within the albuminuria-defined subgroups, a significant positive association between pl-Hp and ox-LDL was observed in both mildly elevated and lower-normal s-ACR ranges. In the subset with 0.2714 < s-ACR < 0.3649 mg/mmol, indicative of early glomerular damage, the association was significant (β = 1.000, *p* < 0.041), reflecting oxidative stress and endothelial dysfunction. Interestingly, this association persisted and was even more pronounced in the s-ACR < 0.2714 mg/mmoL group (β = 2.304, *p* < 0.005), suggesting that pl-Hp and ox-LDL elevations may precede overt albuminuria and serve as early markers of vascular oxidative burden. Unlike s-ACR, cohort stratification based on u-ACR did not show any significant link between pl-Hp and ox-LDL, indicating that the difference may be due to compartmental variations. Both Hp and ox-LDL are circulating markers of systemic inflammation and oxidative stress, which are more closely related to blood-based indices like s-ACR. At the same time, u-ACR primarily reflects renal-specific processes such as glomerular permeability and may not capture systemic oxidative burden, thus weakening its correlation with plasma markers. Although it is well-accepted that u-ACR is a clinically validated predictor for CVD [64,65], a recent study found that a low s-ACR was associated with a poor prognosis in heart failure, especially in patients not using β-blockers and with normal kidney function (Serum Cre < 97 μmol/L) [66]. These findings support the view that oxidative injury can occur before significant glomerular leakage becomes clinically apparent, emphasizing the need for more sensitive biomarkers of early pathophysiological change [67].

Furthermore, participants with elevated IL-6 (IL-6 ≥ 32.9315 pg/mL), a potent proinflammatory cytokine, exhibited a strong positive association between pl-Hp and ox-LDL (β = 1.037, *p* < 0.005). IL-6 is a principal driver of hepatic acute-phase response and has been implicated in upregulating Hp synthesis [68]. This suggests that inflammation may mediate the linkage between lipid peroxidation and acute-phase reactants, forming a vicious cycle that propagates vascular injury and metabolic derangement [69].

Subgroup analysis suggests that the relationship between pl-Hp and ox-LDL is not consistent across all individuals, but somewhat varies depending on renal function, albuminuria, and systemic inflammation. This layered effect underscores the importance of considering patient-specific biological factors when evaluating Hp along with biomarkers of oxidative stress and inflammation. Dividing a relatively small group (N = 57) into quartiles or multiple range-based categories resulted in small cell sizes (approximately 13–28 per quartile), which might have limited the statistical power of stratified analyses and caused potentially unstable estimates and wide confidence intervals. Therefore, the stratified results are presented here as exploratory, should be interpreted cautiously, and require confirmation in larger cohorts. Overall, the clinical implications of these findings are twofold. First, they support the use of pl-Hp as a potential biomarker of systemic oxidative stress in obese T2DM populations, where ox-LDL-mediated vascular injury is highly relevant. Second, they suggest that u-Hp may have limited utility in capturing oxidative stress dynamics related to lipoprotein oxidation, and that its clinical significance may lie elsewhere, specifically in the assessment of renal pathology.

The study has several limitations. First, the relatively small sample size (N = 57), particularly in some subgroups, limits statistical power and may result in unstable estimates; therefore, these findings should be considered exploratory and require validation in larger, independent cohorts. Additionally, it prevents causal inference; hence, prospective studies are needed to determine whether pl-Hp predicts changes in ox-LDL or related cardiovascular outcomes. Second, Hp genotyping was not performed, although Hp polymorphisms are known to influence antioxidant function and may modify associations with oxidative biomarkers. Future studies incorporating Hp genotyping are necessary to clarify whether the associations are influenced by genetic background or reflect absolute pl-Hp levels. Third, although we adjusted for a broad panel of clinical and metabolic covariates, including inflammatory cytokines and adipokines, other untested residual confounders may still be present. Fourth, medication use is an essential potential confounder in studies of oxidative stress and inflammation. In our cohort, a high proportion of patients were receiving statins, antihypertensives, and insulin therapy, all of which may directly or indirectly modulate the biomarkers investigated, including Hp and ox-LDL. However, the diversity in drug classes, dosages, and combinations, coupled with the limited sample size, precluded reliable adjustment for medication use in multivariate models without overfitting. As such, we cannot exclude residual confounding by treatment effects. Future studies with larger cohorts and harmonized medication regimens are needed to delineate the extent to which pharmacological therapy influences the observed associations. Finally, a key limitation of our study is the lack of a control group that is neither diabetic nor obese. Without a comparator population, interpreting whether the observed associations between Hp and ox-LDL are disease-specific or reflect broader physiological processes is constrained. Future studies including matched controls are necessary to determine better how specific these associations are to obese individuals with T2DM. To mitigate this, we systematically compared our data to well-established clinical reference ranges reported from accredited laboratories and published studies. Relying on published reference values range data offers context for interpreting our findings but should not be regarded as definitive normal values. Future longitudinal prospective studies, including well-matched control groups, are necessary to validate and better understand these findings.

## 5. Conclusions

Although both u-Hp and pl-Hp have been linked to cardiovascular outcomes, pl-Hp has often been considered a more reliable marker in obese individuals with T2DM. In our study, pl-Hp showed a positive association with ox-LDL; however, this relationship was attenuated after adjustment for inflammatory and metabolic covariates, suggesting that the link may be mediated by shared pathways of inflammation and adipokine/cytokine activity rather than being independent. These findings indicate that while pl-Hp may reflect systemic processes relevant to cardiovascular risk, its predictive value as a standalone biomarker remains uncertain. Prospective longitudinal studies are required to clarify whether pl-Hp provides independent prognostic information beyond established inflammatory and metabolic markers. Understanding the precise mechanisms linking Hp, oxidative stress, and lipid metabolism will also be critical to evaluating its potential incorporation into cardiometabolic risk models and guiding the development of standardized protocols for its use in clinical practice.

## Figures and Tables

**Table 1:** Baseline characteristics of patient population.

Category	Variable	T2DM Cohort
Demographics	Age (years) *	60.96 ± 9.99
	BMI (kg/m^2^) *	35.15 ± 6.65
Clinical Parameters	Systolic BP (mmHg)	145.00 (123.50–153.50)
	Diastolic BP (mmHg)	71.00 (61.50–81.00)
	HbA1c (%) *	8.66 ± 1.60
	Fasting Glucose (mmol/L)	8.00 (6.67–14.27)
Renal Function & Protein Status	Creatinine (µmol/L)	111.50 (81.00–142.25)
	eGFR (mL/min/1.73 m^2^) *	58.04 ± 26.50
	Serum Albumin (g/L)	41.00 (38.00–43.00)
	Total Protein (g/L)	70.00 (67.50–73.50)
	Hemoglobin (g/L)	120.00 (108.00–130.00)
Lipid Profile	Total Cholesterol (mg/dL)	154.68 (116.01–193.35)
	LDL-c (mg/dL)	77.34 (77.34–116.01)
	HDL-c (mg/dL)	38.67 (38.67–38.67)
	Triglycerides (mg/dL)	177.14 (88.57–177.14)
	ApoB (mg/mL)	86.00 (78.00–103.50)
	Ox-LDL (ng/mL) *	247.97 ± 67.50
Liver Enzymes	AST (U/L)	19.00 (13.00–26.00)
	ALT (U/L)	2.00 (1.00–4.00)
	ALP (U/L)	85.00 (61.00–105.00)
Inflammatory & Adipokine Markers	Hs-CRP (mg/L)	7.60 (3.40–25.97)
	IL-6 (pg/mL)	24.72 (16.25–32.93)
	TNFα (pg/mL)	38.80 (32.01–52.55)
	Adiponectin (ng/mL) *	3735.74 ± 966.21
	Resistin (pg/mL)	46,889.39 (33,574.75–66,234.11)
	Adipsin (pg/mL)	583,813.15 (225,134.29–1,204,432.24)
Urinary Markers	Urine Albumin (mg/L)	16.11 (1.80–85.96)
	Urine Haptoglobin (ng/mL)	10.50 (3.58–73.13)
	Plasma Haptoglobin (ng/mL)	4,105,900 (2,456,100–5,906,600)
	Urine Albumin-to-Creatinine Ratio (u-ACR, mg/mmol)	190.00 (19.95–796.00)
	Serum Albumin-to-Creatinine Ratio (s-ACR, mg/mmol)	0.365 (0.271–0.471)
Family Disease History	Diabetes (%)	81.80
	Hypertension (%)	25.50
	CAD (%)	38.20
	Hypercholesterolemia (%)	57.70
	Stroke (%)	14.30
Medication	Antidiabetic Drugs (%)	
	Insulin	47.37
	Metformin	14.03
	DPP4 inhibitors	14.03
	Sulfonylurea	10.53
	Cardiovascular/Antihypertensive Drugs (%) HMG-CoA Reductase Inhibitors (Statins)	19.30
	Calcium Channel Blockers	8.77
	ACE Inhibitors	7.02
	Diuretics	3.51
	Other Medications (%) NSAID	7.02
	Proton Pump Inhibitors (PPI)	3.51

Data are presented for continuous variables as mean (standard deviation, SD) or median (interquartile range, IQR) and as frequencies (percentages, %) for categorical variables. *, values are normally distributed. Abbreviations: BMI, body mass index; BP, blood pressure; Hb, hemoglobin; AST, aspartate aminotransferase; ALT, alanine aminotransferase; ALP, alkaline phosphatase; HbA1c, hemoglobin A1c; LDL-c, low-density lipoprotein cholesterol; HDL-c, high-density lipoprotein cholesterol; Hs-CRP, high-sensitivity C-reactive protein; eGFR, estimated glomerular filtration rate; ApoB, apolipoprotein B; Ox-LDL, oxidized low-density lipoprotein; IL-6, interleukin 6; TNFα, tumor necrosis factor alpha; Hp, haptoglobin; CAD, coronary artery disease; DPP4, dipeptidyl-peptidase 4; ACE, angiotensin-converting enzyme; NSAID, non-steroidal anti-inflammatory drugs; PPI, proton-pump inhibitor. Lab Corp reference ranges in healthy individuals are: PL-Hp (300,000–2,000,000 ng/mL), u-Hp (70–160 ng/mL), and ox-LDL (10–170 ng/mL). These values should not be regarded as definitive clinical reference standards and must be interpreted with caution, taking into account assay variability and laboratory-specific methodologies.

**Table 2:** Heatmap Spearman correlation matrix between plasma and urinary haptoglobin and ox-LDL, clinical, and metabolic parameters in T2DM patients.

		U-Hp	Pl-Hp	Age	Gender	SBP	DBP	A1C	CRE	ALB	AST	ALT	CHOL	LDL	HDL	TAG	CRP	BMI	ApoB	Ox-LDL	e-GFR	IL-6
U-Hp	R	1	−0.145	0.237	0.093	−0.081	−0.091	0.298 *	0.012	−0.315 *	−0.002	−0.068	0.245	0.254	−0.032	0.265	0.233	−0.043	0.310 *	−0.142	−0.073	0.197
	P	.	0.290	0.082	0.499	0.559	0.508	0.030	0.931	0.023	0.989	0.655	0.109	0.096	0.836	0.086	0.123	0.757	0.021	0.299	0.599	0.150
Pl-Hp	R	−0.145	1	0.182	−0.047	0.176	0.006	−0.223	−0.004	0.042	0.004	−0.100	−0.261	−0.246	0.093	−0.041	−0.364 *	−0.242	−0.063	0.358 **	−0.085	0.025
	P	0.290	.	0.174	0.728	0.190	0.966	0.101	0.976	0.761	0.977	0.504	0.080	0.099	0.541	0.787	0.013	0.070	0.642	0.006	0.535	0.851
Age	R	0.237	0.182	1	−0.076	−0.103	−0.335 *	0.044	0.186	0.027	−0.064	−0.103	−0.191	−0.241	0.042	−0.047	0.013	−0.063	−0.052	0.082	−0.384 **	0.187
	P	0.082	0.174	.	0.574	0.446	0.011	0.751	0.170	0.848	0.668	0.490	0.204	0.107	0.783	0.759	0.933	0.642	0.703	0.543	0.003	0.164
Gender	R	0.093	−0.047	−0.076	1	0.042	−0.198	−0.023	−0.429 ***	−0.022	−0.114	0.012	0.116	0.360 *	−0.060	−0.292	0.053	0.167	−0.002	0.064	0.228	0.116
	P	0.499	0.728	0.574	.	0.758	0.139	0.865	0.0001	0.877	0.444	0.938	0.443	0.014	0.697	0.051	0.727	0.214	0.987	0.635	0.091	0.392
SBP	R	−0.081	0.176	−0.103	0.042	1	0.549 ***	−0.055	0.106	−0.079	0.004	0.194	0.216	0.333 *	−0.155	0.225	0.072	0.070	−0.024	0.077	−0.067	0.089
	P	0.559	0.190	0.446	0.758	.	0.0001	0.692	0.436	0.570	0.979	0.190	0.150	0.024	0.310	0.138	0.634	0.607	0.860	0.571	0.622	0.510
DBP	R	−0.091	0.006	−0.335 *	−0.198	0.549 ***	1	−0.087	−0.004	0.062	0.083	0.085	0.317 *	0.251	−0.262	0.048	−0.011	−0.098	0.040	0.094	0.183	−0.023
	P	0.508	0.966	0.011	0.139	0.0001	.	0.527	0.974	0.655	0.578	0.570	0.032	0.093	0.082	0.755	0.943	0.470	0.767	0.489	0.176	0.864
A1C	R	0.298	−0.223	0.044	−0.023	−0.055	−0.087	1	−0.003	−0.176	0.176	−0.195	0.062	0.100	0.178	0.063	0.147	0.204	0.350 **	−0.098	0.028	0.057
	P	0.030	0.101	0.751	0.865	0.692	0.527	.	0.981	0.211	0.242	0.195	0.683	0.510	0.242	0.681	0.340	0.135	0.009	0.478	0.843	0.680
CRE	R	0.012	−0.004	0.186	−0.429 ***	0.106	−0.004	−0.003	1	−0.322 *	0.029	0.033	−0.089	−0.113	−0.134	0.091	0.083	−0.166	−0.126	0.099	−0.864 ***	0.009
	P	0.931	0.976	0.170	0.0001	0.436	0.974	0.981	.	0.018	0.846	0.830	0.555	0.456	0.382	0.551	0.583	0.221	0.355	0.466	0.0001	0.946
ALB	R	−0.315 *	0.042	0.027	−0.022	−0.079	0.062	−0.176	−0.322 *	1	0.083	0.098	−0.165	−0.369 *	−0.088	−0.028	−0.263	−0.038	−0.161	−0.118	0.429 **	−0.089
	P	0.023	0.761	0.848	0.877	0.570	0.655	0.211	0.018	.	0.581	0.515	0.278	0.013	0.572	0.859	0.084	0.785	0.246	0.395	0.001	0.523
AST	R	−0.002	0.004	−0.064	−0.114	0.004	0.083	0.176	0.029	0.083	1	0.193	0.072	−0.155	−0.303	0.082	−0.142	0.053	0.101	0.180	−0.076	0.232
	P	0.989	0.977	0.668	0.444	0.979	0.578	0.242	0.846	0.581	.	0.194	0.656	0.334	0.057	0.615	0.387	0.722	0.500	0.227	0.616	0.117
ALT	R	−0.068	−0.100	−0.103	0.012	0.194	0.085	−0.195	0.033	0.098	0.193	1	0.161	0.087	−0.232	0.012	−0.146	−0.015	−0.154	−0.125	−0.016	0.021
	P	0.655	0.504	0.49	0.938	0.190	0.570	0.195	0.830	0.515	0.194	.	0.315	0.587	0.150	0.941	0.374	0.921	0.302	0.401	0.916	0.888
CHOL	R	0.245	−0.261	−0.191	0.116	0.216	0.317 *	0.062	−0.089	−0.165	0.072	0.161	1	0.661 ***	−0.253	0.297 *	0.056	0.091	0.446 **	−0.048	0.165	0.086
	P	0.109	0.08	0.204	0.443	0.150	0.032	0.683	0.555	0.278	0.656	0.315	.	0.0001	0.094	0.047	0.739	0.546	0.002	0.752	0.274	0.569
LDL	R	0.254	−0.246	−0.241	0.360 *	0.333 *	0.251	0.100	−0.113	−0.369 *	−0.155	0.087	0.661 ***	1	−0.107	0.083	0.081	0.061	0.553 ***	−0.032	0.173	−0.059
	P	0.096	0.099	0.107	0.014	0.024	0.093	0.510	0.456	0.013	0.334	0.587	0.0001	.	0.484	0.588	0.629	0.685	0.0001	0.833	0.251	0.699
HDL	R	−0.032	0.093	0.042	−0.060	−0.155	−0.262	0.178	−0.134	−0.088	−0.303	−0.232	−0.253	−0.107	1	0.064	0.219	−0.236	0.218	−0.079	0.165	0.038
	P	0.836	0.541	0.783	0.697	0.310	0.082	0.242	0.382	0.572	0.057	0.150	0.094	0.484	.	0.682	0.194	0.119	0.151	0.607	0.280	0.803
TAG	R	0.265	−0.041	−0.047	−0.292	0.225	0.048	0.063	0.091	−0.028	0.082	0.012	0.297 *	0.083	0.064	1	0.165	0.109	0.233	−0.206	0.011	0.157
	P	0.086	0.787	0.759	0.051	0.138	0.755	0.681	0.551	0.859	0.615	0.941	0.047	0.588	0.682	.	0.328	0.477	0.123	0.176	0.942	0.303
CRP	R	0.233	−0.364 *	0.013	0.053	0.072	−0.011	0.147	0.083	−0.263	−0.142	−0.146	0.056	0.081	0.219	0.165	1	0.260	0.042	−0.149	−0.141	0.181
	P	0.123	0.013	0.933	0.727	0.634	0.943	0.340	0.583	0.084	0.387	0.374	0.739	0.629	0.194	0.328	.	0.081	0.781	0.324	0.350	0.230
BMI	R	−0.043	−0.242	−0.063	0.167	0.07	−0.098	0.204	−0.166	−0.038	0.053	−0.015	0.091	0.061	−0.236	0.109	0.260	1	−0.141	−0.119	0.107	0.053
	P	0.757	0.070	0.642	0.214	0.607	0.470	0.135	0.221	0.785	0.722	0.921	0.546	0.685	0.119	0.477	0.081	.	0.297	0.376	0.434	0.695
ApoB	R	0.310 *	−0.063	−0.052	−0.002	−0.024	0.040	0.350 **	−0.126	−0.161	0.101	−0.154	0.446 **	0.553 ***	0.218	0.233	0.042	−0.141	1	−0.130	0.180	0.043
	P	0.021	0.642	0.703	0.987	0.860	0.767	0.009	0.355	0.246	0.500	0.302	0.002	0.0001	0.151	0.123	0.781	0.297	.	0.336	0.185	0.749
Ox-LDL	R	−0.142	0.358 **	0.082	0.064	0.077	0.094	−0.098	0.099	−0.118	0.180	−0.125	−0.048	−0.032	−0.079	−0.206	−0.149	−0.119	−0.130	1	−0.241	0.340 *
	P	0.299	0.006	0.543	0.635	0.571	0.489	0.478	0.466	0.395	0.227	0.401	0.752	0.833	0.607	0.176	0.324	0.376	0.336	.	0.074	0.010
e-GFR	R	−0.073	−0.085	−0.384 **	0.228	−0.067	0.183	0.028	−0.864 ***	0.429 **	−0.076	−0.016	0.165	0.173	0.165	0.011	−0.141	0.107	0.180	−0.241	1	−0.147
	P	0.599	0.535	0.003	0.091	0.622	0.176	0.843	0.0001	0.001	0.616	0.916	0.274	0.251	0.280	0.942	0.350	0.434	0.185	0.074	.	0.279
IL−6	R	0.197	0.025	0.187	0.116	0.089	−0.023	0.057	0.009	−0.089	0.232	0.021	0.086	−0.059	0.038	0.157	0.181	0.053	0.043	0.340 *	−0.147	1
	P	0.150	0.851	0.164	0.392	0.510	0.864	0.680	0.946	0.523	0.117	0.888	0.569	0.699	0.803	0.303	0.230	0.695	0.749	0.010	0.279	.
						Heatmap scale:		−1	−0.75	−0.50	−0.25	0	0.25	0.50	0.75	1						

Results are expressed as Spearman’s Rho coefficient (R) for 2-tailed Spearman correlation analysis. Colors in the heatmap show the strength and direction of rho correlation coefficients, with red representing positive correlations and blue representing negative correlations. The color intensity indicates the size of the coefficient, making it easier to compare variables visually. Abbreviations: U-Hp, Urinary Haptoglobin; Pl-Hp, Plasma Haptoglobin; SBP, Systolic blood pressure; DBP, Diastolic blood pressure; A1C, Hemoglobin A1c; CRE, Creatinine; ALB, Albumin; CHOL, Total cholesterol; LDL, low-density lipoprotein cholesterol; HDL, high-density lipoprotein cholesterol; TAG, Triglycerides; BMI, Body mass index; CRP, high sensitivity C-reactive protein; e-GFR, estimated glomerular filtration rate; IL-6, Interleukin 6; ApoB, apolipoprotein B; Ox-LDL, oxidized low-density lipoprotein. Statistical significance: * *p* < 0.05; ** *p* < 0.01; and *** *p* < 0.001.

**Table 3:** Univariate regression analysis between urinary and pl-Hp and ox-LDL in T2DM patients.

Dependent Variable: Ox-LDL		Unstandardized Coefficients		Standardized Coefficients	t	*p*		95% CI for B	
		B	SE	B				Lower Bound	Upper Bound
	(Constant)	244.222	10.690		22.846	0.0001		222.781	265.663
	**U-Hp**	0.038	0.069	0.075	0.549	**0.585**		−0.101	0.177
		Unstandardized Coefficients		Standardized Coefficients	t	*p*	95% CI for B		
		B	SE	B			Lower Bound		Upper Bound
	(Constant)	211.000	14.093		14.974	0.0001	182.787		239.272
	**Pl-Hp**	0.741	0.229	0.400	3.237	**0.002**	0.282		1.000

Univariate linear regression analysis was performed to determine the association between u-Hp and pl-Hp. *p* represents the probability value for the regression analysis, with a *p* value ≤ 0.05 being considered significant. CI denotes the confidence interval for the beta (B) coefficient. The predictors (independent variables) are u-Hp or pl-Hp, while the dependent variable (DV) is ox-LDL. “(Constant)” refers to the intercept of the regression equation. It represents the predicted value of the dependent variable when all independent variables are equal to zero.

**Table 4:** Multi-regression analysis for the association of pl-Hp with ox-LDL in T2DM patients.

Model 1 (R^2^ = 0.158) DV: Ox-LDL		Unstandardized Coefficients		*p*	95% CI for B	
		B	SE		Lower Bound	Upper Bound
	(Constant)	205.949	18.343	0.0001	168.487	243.411
	**Pl-Hp**	0.632	0.267	**0.024**	0.087	1.177
Model 2 (R^2^ = 0.481) DV: Ox-LDL		Unstandardized Coefficients		*p*	95% CI for B	
		B	SE		Lower Bound	Lower Bound
	(Constant)	48.575	111.405	0.666	−180.009	277.160
	**Pl-Hp**	0.614	0.281	**0.038**	0.037	1.191
	Age	1.988	1.287	0.134	−0.653	4.629
	Gender	10.968	23.666	0.647	−37.590	59.527
	HbA1c	2.099	6.768	0.759	−11.787	15.986
Model 3 (R^2^ = 0.722) DV: Ox-LDL		Unstandardized Coefficients		*p*	95% CI for B	
	(Constant)	B	SE		Lower Bound	Upper Bound
		−330.775	217.205	0.149	−793.736	132.186
	**Pl-Hp**	0.678	0.267	**0.023**	0.108	1.248
	Age	1.702	1.207	0.179	−0.872	4.275
	Gender	47.144	25.183	0.081	−6.532	100.821
	A1c	4.438	8.425	0.606	−13.519	22.395
	Alb	3.427	2.492	0.189	−1.885	8.738
	AST	0.536	0.898	0.560	−1.377	2.449
	ALT	−4.091	2.598	0.136	−9.629	1.446
	Chol-t	68.862	25.024	0.015	15.526	122.199
	LDL-c	−43.162	27.887	0.143	−102.601	16.278
	HDL-c	44.797	33.949	0.207	−27.563	117.158
	TAG	−46.197	15.593	0.010	−79.432	−12.962
	SBP	−0.375	0.709	0.605	−1.885	1.136
	DBP	1.189	1.512	0.444	−2.034	4.411
	BMI	−0.247	2.502	0.923	−5.580	5.087
	CRE	0.293	0.138	0.052	−0.002	0.588
	Hs-CRP	−0.568	0.354	0.130	−1.323	0.187
Model 4 (R^2^ = 0.636) DV: Ox-LDL (Constant)		Unstandardized Coefficients		*p*	95% CI for B	
		B	SE		Lower Bound	Upper Bound
		275.371	67.129	0.000	138.796	411.945
**Pl-Hp**		0.028	0.020	**0.177**	−0.013	0.069
Age		0.702	0.729	0.343	−0.782	2.186
Gender		−7.756	15.163	0.612	−38.606	23.095
A1c		−3.986	4.380	0.369	−12.897	4.925
Hs-CRP		−0.542	0.217	0.018	−0.984	−0.101
IL-6		−0.034	0.751	0.964	−1.562	1.493
TNFα		0.554	0.628	0.384	−0.723	1.830
Adiponectin		−0.556	0.127	0.000	−0.814	−0.298
Resistin		0.000	0.000	0.047	0.000	0.001
Adipsin		0.000	0.000	0.466	0.000	0.000

Multiple regression analysis was conducted for the dependent variable (DV): ox-LDL. The predictors (independent variables) include plasma Haptoglobin (Pl-Hp) and other variables, such as age, gender, HbA1C, total cholesterol, LDL-c, HDL-c, triglycerides, hs-CRP, BMI, and relevant cytokines and adipokines. R^2^ indicates the proportion of variance in the dependent variable (DV) explained by the independent variable in each linear regression model (M1-M3). *p* represents the probability value for each independent variable in the regression models. A *p* value ≤ 0.05 is considered significant. CI refers to the confidence interval for the beta (B) coefficient. “(Constant)” refers to the intercept of the regression equation. It represents the predicted value of the dependent variable when all independent variables are equal to zero.

**Table 5:** Stepwise regression analysis for the association between pl-Hp and ox-LDL in T2DM patients.

Model 1 (R^2^ = 0.174) DV: Ox-LDL		Unstandardized Coefficients		*p*	95% CI for B	
M1	(Constant)	B	SE		Lower Bound	Upper Bound
		212.899	15.957	0.0001	180.698	245.101
	**Pl-Hp**	0.736	0.248	**0.005**	0.237	1.236
Model 2 (R^2^ = 0.269) DV: Ox-LDL		Unstandardized Coefficients		*p*	95% CI for B	
M2	(Constant)	B	SE		Lower Bound	Upper Bound
		172.918	23.074	0.0001	126.320	219.516
	**Pl-Hp**	0.680	0.237	**0.006**	0.202	1.159
	IL-6	1.593	0.692	0.026	0.196	2.990

Stepwise regression analysis was conducted for the dependent variable (DV), ox-LDL. The predictors (independent variables) included hs-CRP, IL-6, eGFR, u-ACR, and s-ACR, as well as plasma haptoglobin (pl-Hp) in the model. R^2^ indicates the proportion of variance in the dependent variable (DV) explained by the independent variable in each linear regression model (M1-M2). *p* represents the probability value for each independent variable in the regression models. A *p*-value of ≤ 0.05 is considered significant. CI refers to the confidence interval for the beta (B) coefficient. “(Constant)” refers to the intercept of the regression equation. It represents the predicted value of the dependent variable when all independent variables are equal to zero.

**Table 6:** Association between pl-Hp and ox-LDL across stratified groups of inflammatory and kidney function biomarkers.

**Independent Variable**		**Unstandardized** **Coefficients**		***p***	**95% CI for B**	
e-GFR * mL/min/1.73 m^2^	Categories	B	SE		Lower Bound	Upper Bound
	e-GFR ≥ 60 (N = 23 ), R^2^ = 0.094	0.413	0.279	0.154	−0.168	0.994
	e-GFR = 30–59 (N = 25), R^2^ = 0.310	1.318	0.384	0.002	0.523	2.112
	e-GFR < 30 (N = 8), R^2^ = 0.437	2.173	0.810	0.031	0.259	4.087
u-ACR mg/mmoL	u-ACR ≤ 19.95 (N = 13), R^2^ = 0.000	−0.028	3.061	0.993	−6.698	6.642
	19.95 < u-ACR < 190.00 (N = 14), R^2^ = 0.003	0.050	0.235	0.836	−0.457	0.557
	u-ACR ≥ 190 (N = 28), R^2^ = 0.039	−0.011	0.011	0.306	−0.033	0.011
s-ACR mg/mmoL	s-ACR < 0.2714 (N = 14), R^2^ = 0.490	2.304	0.678	0.005	0.826	3.781
	0.2714 < s-ACR < 0.3649 (N = 15), R^2^ = 0.285	1.000	0.447	0.041	0.051	1.000
	0.3649 < s-ACR < 0.4707 (N = 15), R^2^ = 0.067	0.506	0.526	0.353	−0.630	1.001
	s-ACR ≥ 0.4707 (N = 13), R^2^ = 0.178	0.505	0.327	0.151	−0.215	1.000
IL-6 pg/mL	IL-6 < 16.2481 (N = 14), R^2^ = 0.490	0.209	0.737	0.781	−1.395	1.814
	16.2481 < IL-6 < 24.7162 (N = 14), R^2^ = 0.285	0.813	0.501	0.131	−0.280	1.905
	24.7162 < IL-6 < 32.9315 (N = 15), R^2^ = 0.067	0.481	0.421	0.274	−0.428	1.391
	IL-6 ≥ 32.9315 (N = 14), R^2^ = 0.178	1.037	0.302	0.005	0.378	1.695

Multiple regression analysis was conducted to explore the association between the dependent variable (ox-LDL) and the independent variable, plasma haptoglobin (Pl-Hp). The regression analysis was performed among categorized groups (quartiles: Q1–Q4) for the random urine albumin-to-creatinine ratio (u-ACR), serum albumin-to-creatinine ratio (s-ACR), and interleukin-6 (IL-6). e-GFR values were categorized into three main groups that align with ranges based on KDIGO (Kidney Disease Improving Global Outcomes) guidelines: Normal or mildly decreased kidney function (e-GFR ≥ 60), Moderate decrease in kidney function (e-GFR = 30–59), and Severe decrease to Kidney Failure (e-GFR < 30). *, indicates one missing eGFR value not recorded. R^2^ represents the proportion of variance in the dependent variable (DV) explained by the independent variable in the linear regression model. *p* refers to the probability value for each independent variable included in the regression models. A *p*-value ≤ 0.05 is considered statistically significant. CI denotes the confidence interval for the beta (B) coefficient.

## Data Availability

All data presented are included in the manuscript.

## Abbreviations

The following abbreviations are used in this manuscript:

ALP	Alkaline phosphatase
ALT	Alanine aminotransferase
ApoB	Apolipoprotein B
AST	Aspartate aminotransferase
BMI	Body mass index
Cre	Creatinine
CVD	Cardiovascular disease
EGFR	Estimated glomerular filtration rate
Hb	Hemoglobin
HbA1c	Glycated Hemoglobin A1c
HDL	High-density lipoprotein
Hp	Haptoglobin
Hs-CRP	High-sensitivity C-reactive protein
IL-6	Interleukin 6
LDL	Low-density lipoprotein
Ox-LDL	Oxidized low-density lipoprotein
Pl-Hp	Plasma haptoglobin
S-ACR	Serum albumin-to-creatinine ratio
T2DM	Type 2 diabetes mellitus
U-ACR	Urinary albumin-to-creatinine ratio
U-Hp	Urinary haptoglobin
